# Priorities for protecting health from climate change in the WHO European Region: recent regional activities

**DOI:** 10.1007/s00103-019-02943-9

**Published:** 2019-04-18

**Authors:** Vladimir Kendrovski, Oliver Schmoll

**Affiliations:** World Health Organization Regional Office for Europe, European Centre for Environment and Health, Platz der Vereinigten Nationen 1, 53113 Bonn, Germany

**Keywords:** Adaptation, Greenhouse gases, Public health, Mitigation, World Health Organization, Anpassung, Treibhausgas, Gesundheitswesen, Klimaschutzmaßnahmen, Weltgesundheitsorganisation

## Abstract

Evidence of the impact of climate change on health is growing. Health systems need to be prepared and gradually adapt to the effects of climate change, including extreme weather events.

Fossil fuel combustion as the driver of climate change poses a tremendous burden of disease. In turn, cutting greenhouse gas emissions in all sectors will achieve health co-benefits. If all countries meet the Paris Agreement by 2030, the annual number of avoidable premature deaths could total 138,000 across the entire European Region of the World Health Organization (WHO).

Several international frameworks promote a stronger commitment by countries to implementing the necessary adaptations in the health sector and to addressing health considerations in adaptation measures in other sectors. The WHO has a mandate from its member states to identify solutions and help prevent or reduce health impacts, including those from climate change.

National governments are continuing to establish public health adaptation measures, which provide a rationale for and trigger action on climate change by the health community. Effective national responses to climate risks require strategic analyses of current and anticipated threats. Health professionals need to play a proactive role in promoting health arguments and evidence in the formulation of national climate change adaptation and mitigation responses. To this end, country capacities need to be further strengthened to identify and address local health risks posed by climate change and to develop, implement and evaluate health-focused interventions through integrated approaches. Building climate-resilient and environmentally sustainable health care facilities is an essential pillar of health sector leadership to address climate change.

## Zusammenfassung

Es gibt immer mehr Erkenntnisse zu den Auswirkungen des Klimawandels auf die Gesundheit. Die Gesundheitssysteme müssen darauf vorbereitet werden und sich schrittweise an die Auswirkungen des Klimawandels einschließlich extremer Wetterereignisse anpassen. Von der Verbrennung fossiler Brennstoffe als treibender Faktor des Klimawandels geht eine große Krankheitslast aus. Umgekehrt wird die Senkung der Treibhausgasemissionen in allen Sektoren auch mit positiven Nebeneffekten für die Gesundheit einhergehen. Wenn alle Länder bis 2030 das Pariser Übereinkommen erfüllen, könnte sich die jährliche Zahl der vermeidbaren vorzeitigen Todesfälle in der gesamten europäischen Region der Weltgesundheitsorganisation (WHO) auf 138.000 belaufen. Mehrere internationale Rahmenbedingungen fördern ein stärkeres Engagement der Länder für die Umsetzung notwendiger Anpassungen im Gesundheitssektor und für die Berücksichtigung gesundheitspolitischer Aspekte bei Anpassungsmaßnahmen anderer Sektoren. Die WHO hat von ihren Mitgliedstaaten den Auftrag, Lösungen zu benennen und zur Vermeidung oder Verringerung negativer gesundheitlicher Auswirkungen, auch durch den Klimawandel, beizutragen. Die nationalen Regierungen führen weiterhin Anpassungen im Bereich der öffentlichen Gesundheit ein, die eine Begründung für Maßnahmen der Gesundheitsgemeinschaft gegen den Klimawandel bieten und solche Maßnahmen auslösen. Wirksame innerstaatliche Reaktionen auf Klimarisiken erfordern strategische Analysen aktueller und erwarteter Bedrohungen. Die Angehörigen der Gesundheitsberufe müssen eine proaktive Rolle spielen, indem sie die stärkere Berücksichtigung gesundheitlicher Argumente und Erkenntnisse bei der Formulierung innerstaatlicher Maßnahmen zur Anpassung an den Klimawandel und zum Klimaschutz fördern. Hierzu müssen die Kapazitäten der Länder weiter ausgebaut werden, damit von Klimaänderungen ausgehende lokale Gesundheitsrisiken identifiziert werden können, damit diesen entgegengewirkt werden kann und damit durch integrierte Ansätze gesundheitsförderliche Maßnahmen entwickelt, durchgeführt und bewertet werden können. Der Aufbau klimaresistenter und ökologisch nachhaltiger Gesundheitseinrichtungen ist eine wesentliche Säule der Führungsrolle des Gesundheitssektors bei der Bekämpfung des Klimawandels.

## Introduction

Some of the climatic change in recent years has established new record levels, such as for global and European temperatures, winter Arctic sea ice extent and sea levels [1]. Climate change is already affecting human health, with increasing exposures and vulnerability recorded worldwide [2].

Key reports of global and European relevance include the series of government-approved reports from the Intergovernmental Panel on Climate Change (IPCC), specifically the special report on the impacts of global warming of 1.5 °C [2] and its fifth assessment report, which reviewed the evidence on climate change and health and provided summaries for policy-makers [3, 4]. The health synthesis report aims to summarize the findings of the IPCC special 1.5 report regarding the relationship between climate change and health [5]. In 2015, the Lancet Commission published the report on climate change and global environmental change [6]. In 2017, the WHO Regional Office for Europe presented an update on protecting health in Europe from climate change, drawing on the extensive body of new research and evidence [7].

Health impacts of climate change and variability are being observed: direct impacts result through temperature increases, heat waves, storms, forest fires, floods and droughts. Indirect impacts are mediated through the effects of climate change on biodiversity, vectors distribution, allergens, ecosystems and productive sectors, such as agriculture, water and food supplies.

Climate change will affect everybody, but vulnerability to weather and climate change depends on people’s level of exposure, their personal characteristics (such as age, education, income and health status) and their access to health services. Elderly people, children, outdoor workers and homeless people are particularly susceptible population groups [8, 9]. The effects of exposure can be direct or indirect, for example heat spells may directly cause heat stress, dehydration or heat stroke, while the worsening of cardiovascular and respiratory conditions or electrolyte disorders may be indirect consequences [10, 11]. Climate change affects environmental conditions and social infrastructure, which also determine the health effects, ranging from death to loss of well-being and productivity.

**Fig. 1:**
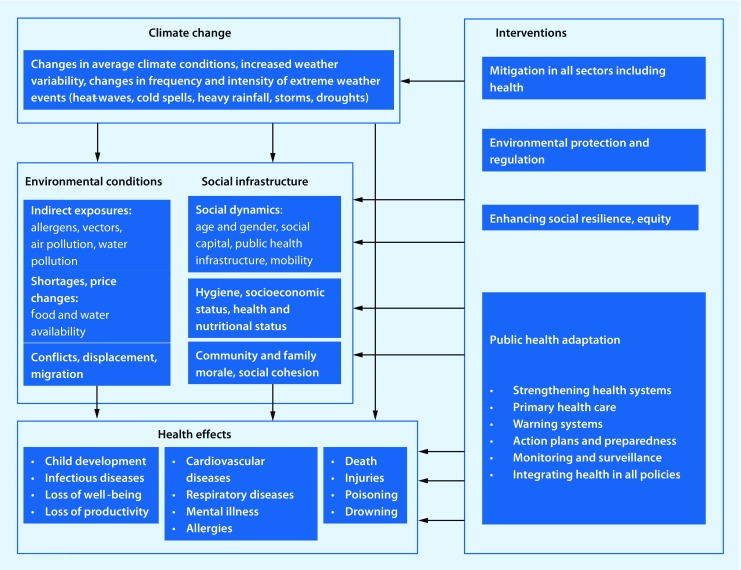
Pathways of climate change and health (adapted from WHO Regional Office for Europe [7])

The pathways by which climate change can affect health have been explained in a number of conceptual frameworks [6, 12]. Fig. 1 presents a combination and adaptation of these frameworks relevant to the WHO European Region [7].

The WHO Regional Office for Europe works with the Member States to generate evidence, develop supporting tools and to identify best policy options to minimize the health effects of climate change. The aim of this article is to support the communication and implementation of existing global and regional commitments and priorities to protect health from the adverse effects of climate change.

## Climate change is a matter of public health

Climate change is influencing mortality, injury and morbidity rates of both communicable (such as vector- and waterborne diseases) and non-communicable (such as cardiovascular and respiratory diseases as well as mental health issues) diseases [6, 7, 9].

The increasing frequency and intensity of extreme weather events due to climate change pose growing risks to human health. Heat-waves were the deadliest extreme climate event in Europe between 1991 and 2015, particularly in southern and western Europe. Several extreme heat-waves have occurred since 2000 (in 2003, 2006, 2007, 2010, 2014, 2015 and 2016) [1]. Exceptionally persistent and high July temperatures in 2018 baked countries across the WHO European Region, including northern Europe and even above the Arctic circle in Lapland, setting the stage for catastrophic forest fires in Greece, for example [13]. Sweden experienced a large number of wildfires due to a prolonged heatwave in summer 2018, which the Swedish Civil Contingencies Agency considered the most serious in the modern history of the country [14]. In the summer of 2017, Portugal was severely affected by wildfires, which occurred during a concurrent heatwave and severe drought, killing 65 people [15]. In Greece, Italy and France, severe alert warning messages were also issued in 2017, indicating that even healthy and active people could suffer from possible negative effects. In Italy, hospital admissions went up by 15% during the heatwave [16]. The heatwave during the summer of 2003 claimed more than 70,000 lives across mostly western European countries [17], and in 2010 many eastern European cities recorded extremely high temperatures, particularly in the Russian Federation, where the deaths attributable to these high temperatures were estimated at around 55,000 [18]. Urban populations are at risk of multiple exposures; for example air pollution also increases the health risks associated with high temperatures [11].

High air temperatures can adversely affect food quality during transport, storage and handling. Elevated marine water temperatures accelerate the growth rate of certain pathogens, such as Vibrio species that can cause foodborne outbreaks after eating seafood and wound infections in injured skin exposed to contaminated marine water [1].

Cold spells were the deadliest weather extremes in eastern Europe, with cumulative numbers of deaths of 28 per 1,000,000 people over the whole time period (1991–2015) [1]. Prolonged cold spells affect physiological and pathological health, especially among elderly people and those with respiratory and cardiovascular diseases [19].

By the end of the 21st century, two thirds of Europeans could be exposed to weather-related disasters every year, compared with only 5% during the period 1981–2010. Climate change is the dominant driver of the projected trends, accounting for more than 90% of the rise in the risk to humans [20]. Flood events registered since 1991 have caused the death of more than 2000 people in the WHO European Region, affected 8.7 million others and generated at least 72 billion Euro in losses [21]. Two thirds of deaths associated with flooding occur from drowning; the rest result from physical trauma, heart attacks, electrocution, carbon monoxide poisoning or fire associated with flooding. Infectious disease vectors such as mosquitoes and rodents may also increase as a result of flooding [1].

Climate change is likely to cause changes in ecological systems that will affect the risk of infectious diseases in the WHO European Region through water, food, air, rodents and arthropod vectors [7, 22]. Waterborne pathogens may be transmitted through two major exposure pathways: drinking water (if water treatment and disinfection are inappropriate) and recreational water use. Heavy precipitation and flooding events can disrupt water treatment and distribution infrastructures, increasing the risk of ingress of faecal pathogens and thus of waterborne outbreaks [21, 22].

Climate risks associated with increases in drought frequency and magnitude include impacts on quality and quantity of freshwater resources, including eutrophication and algae blooms, with possible impacts on drinking-water quality. Droughts may also compromise food safety and security and cause mental health effects, vector-borne diseases and injuries due to lower than usual water levels in lakes and rivers that are used for recreation [23]. Many parts of the Mediterranean region experienced significant drought in 2017, including Italy with the most severe anomalies in annual rainfall 26% below the 1961–1990 average [24]. Water scarcity is accelerating across the European Region and can pose additional challenges for providing sustainable water and sanitation services. The occurrence of waterborne diseases is related to water quality and may be affected by changes in runoff, seasonality and frequency of extreme events such as heavy rains, floods and droughts [12]. Areas under high water stress, for example, are estimated to increase from 19% in 2007 to 35% by the 2070s, by which time the number of additional people affected is expected to reach 16 million to 44 million [7].

## Global and regional policy frameworks for climate action and health

An important aspect of tackling challenges around health and climate change is establishing mechanisms to monitor health impacts and setting targets to reduce these. Several international policy frameworks and platforms are in place (Table 1); these stipulate a clear mandate to foster stronger engagement of the health sector with climate change adaptation and mitigation.

**Table 1:** International policy frameworks for climate action and health

Framework	Description
Paris Agreement on Climate Change under the United Nations Framework Convention on Climate Change (UNFCCC) [25]	The Paris Agreement on Climate Change sets an overall framework for intergovernmental efforts to tackle the challenge posed by climate change. Under the UNFCCC process, it is the first universal, legally binding global deal to combat climate change and adapt to its effects
2030 Agenda for Sustainable Development [26]	This agenda defines 17 sustainable development goals (SDGs) and 169 targets focusing on people, planet, prosperity, peace and partnership. Health is central to the three dimensions (social, environmental and economic) of sustainable development and to measuring its progress. The SDGs support the need for early warning and disaster risk reduction systems, adaptation to climate change, strengthened resilience, adequate facilities and infrastructure and appropriate policies
Sendai Framework for Disaster Risk Reduction 2015–2030 [27]	This framework was adopted by representatives from 187 United Nations Member States in March 2015. As disaster risk reduction generally aims to prevent new and reduce existing disaster risk and to manage residual risk, it contributes to strengthening resilience and therefore to the achievement of sustainable development. Four of the seven Sendai Framework global targets have direct links to health, focusing on reducing mortality, increasing population well-being, improving early warning systems and promoting the safety of health facilities and hospitals
Sixth Ministerial Conference on Environment and Health [28]	The conference took place in Ostrava, Czech Republic, in 2017 and brought together health and environment ministers and high-level representatives of Member States in the WHO European Region. They committed to strengthening and promoting actions to improve the environment and health at the international, national and sub-national levels through the Ostrava Declaration. According to this declaration, by enhancing national implementation, countries will develop national portfolios of action on environment and health by the end of 2018, as standalone policy documents or parts of others, respecting differences in countries’ circumstances, needs, priorities and capacities. The portfolios should draw on Annex 1 to the Declaration, which is a compendium of possible actions to facilitate its implementation, focusing on the seven priority areas, including climate change and health [29]
The Protocol on Water and Health to the Convention on the Protection and Use of Transboundary Watercourses and International Lakes (Water Convention) [30]	Adopted in 2005, the protocol is the first legally binding multilateral agreement to ensure safe drinking water and sanitation in the WHO European Region. Its goal is to protect human health and well-being through improved water resource management and by prevention, control and reduction of water-related diseases, as well as detection, contingency planning and response to outbreaks. A key priority of the protocol’s programme of work is building climate-resilient water and sanitation services
World Health Assembly Resolution WHA61.19 on climate change and health [31]	All Member States in the WHO European Region, including the 28 EU countries, approved Resolution WHA61.19 in 2008, which urges countries to:
	Include health measures in adaptation plans
	Build technical, strategic and leadership capacity in the health sector
	Strengthen capacity for preparedness for and response to natural disasters
	Promote active cross-sectoral engagement in the health sector
	Express commitment to meeting the challenges of climate change, and guide planning and investments
Thirteenth general programme of work 2019–2023 (GPW13) [32]	GPW13 sets out WHO’s strategic direction towards improving the health of the world over the coming 5 years. It highlights the importance of addressing climate change and health, specifically in small island developing states and other vulnerable settings, and of strengthening cross-sectoral collaboration towards health in all policies

Since 1999, with the adoption of the Declaration of the Third Ministerial Conference on Environment and Health held in London, United Kingdom [33], Member States of the WHO European Region have been committed to action towards mitigation of and adaptation to climate change.

## Health in climate change adaptation

Coherent multisectoral action is necessary to effectively tackle the challenges posed by climate change. Health considerations are increasingly on the agendas of sectors and actors addressing climate change. In turn, a consideration of climate change warrants a correspondingly prominent place on the health agenda. The effects of climate change may threaten the overall progress made in reducing the burden of diseases and injuries by increasing morbidity and mortality. Evidence suggests that there is a very high benefit–cost ratio for health adaptation, and that higher benefits are achieved with early adaptation action [34].

Under the UNFCCC process, the Paris Agreement on Climate Change is the first universal, legally binding global deal to combat climate change and adapt to its effects [25]. Its global goal on adaptation focuses on “enhancing adaptive capacity, strengthening resilience and reducing vulnerability to climate change, with a view to contributing to sustainable development and ensuring an adequate adaptation response in the context of the global temperature goal”. With regard to health, implementation of the Paris Agreement provides its parties with opportunities to strengthen the climate resilience of their health systems—for example, through improved disease surveillance and preparedness for extreme weather events and ensuring climate-resilient health facilities, with undisturbed access of health facilities to essential services such as energy, water and sanitation.

With regard to the Paris Agreement, only 18% of all 53 WHO European Member States refer to health in their “intended nationally determined contributions” (INDCs) when outlining commitments to achieving climate-related policy goals and targets, compared with 67% of countries globally [35].

To promote and position health as a key driver for climate actions, the health community needs to play an active role in awareness-raising and advocacy, and in strengthening the evidence base on the health impacts of climate change. This also includes integrating climate resilience into existing and future core health system programming and developing tools to assess the health implications of mitigation policies [36].

**Fig. 2:**
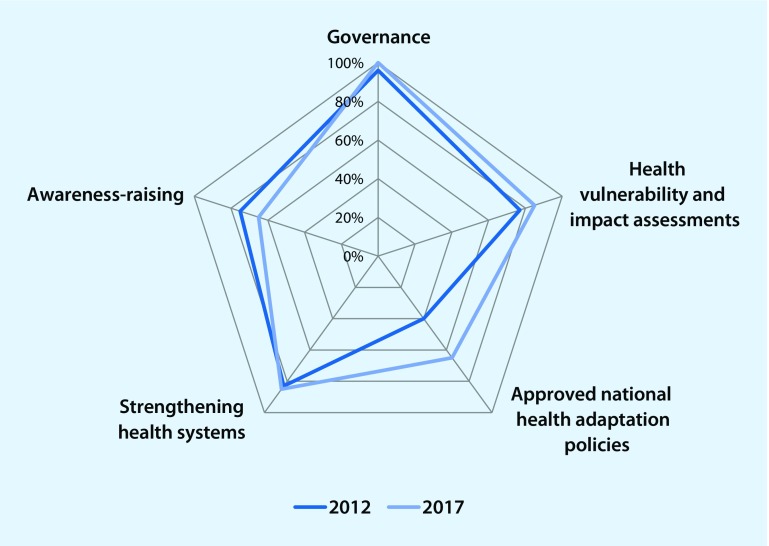
Summary of WHO survey responses by topic in 2012 and 2017 (adapted from Wolf et al. [37] and WHO Regional Office for Europe [38])

The WHO carried out targeted surveys in 2012 and 2017 among its Member States (i. e. with 22 countries participating in 2012 and 20 countries in 2017) to track the status and progress of how health is positioned in existing climate change policies and programming in the European Region [37, 38]. The surveys primarily focused on governance of climate change and health, the status of health vulnerability and impact assessments, the existence of national adaptation health policies, the strengthening of health systems and the raising of awareness. The findings are summarized in Fig. 2.

Governance mechanisms on climate change and health improved between 2012 and 2017. In 2012, already 96% of responding countries had established a multisectoral committee on climate change whose primary role is to coordinate actions and policies for both adaptation and mitigation, including relevant health aspects. In 2017, all responding countries confirmed the existence of such a governmental body.

Similar progress could be observed in implementing health vulnerability and impact assessments. These assessments are a key instrument to provide information for decision-makers on the extent and magnitude of likely health risks attributable to climate change and to identify and prepare for changing health risks. They can suggest priority policies and programmes that can prevent or reduce the severity of future climate change health impacts. WHO developed a guideline that is designed to provide the basics on conducting a national or sub-national health vulnerability and impact assessments [39]. In 2012, 77% of the 22 responding countries stated that they had conducted health-specific assessments of the impacts, vulnerability and adaptation to climate change. In 2017, the percentage of countries performing such assessments had increased to 85% of the 20 responding countries.

Adaptation is defined by the IPCC as “the process of adjustment to actual or expected climate and its effects. In human systems, adaptation seeks to moderate harm or exploit beneficial opportunities. In natural systems, human interventions may facilitate adjustment to expected climate and its effects” [12]. As climate change is one of the many factors associated with the incidence of numerous adverse health outcomes, there is a need to design policies, plans and measures that address the health risks of climate change in order to prevent and reduce the severity of current and future impacts. The development of adaptation plans and programmes for the health sector will depend on and vary according to the specific needs identified during vulnerability impact assessments [40, 41]. In 2012, national health adaptation plans or strategies on climate change had been developed in 64% of the 22 responding countries, with nine countries (40%) reporting that these policies were approved by the government. In 2017, 15 of the responding countries had developed a climate change health adaptation strategy and an associated implementation plan (75%), and these were approved by the government in 13 countries (65%).

In 2012, 83% of responding countries reported that they had taken actions towards strengthening public health capacities and health systems to cope with impacts of climate change; at 85% in 2017, this figure remained almost unchanged. The examples of measures taken by Member States to improve health systems included strengthened early-warning systems and responses, infectious disease surveillance, as well as improved water and sanitation services.

In 2012, 75% of responding Member States reported a high level of awareness of the relevance of health effects on climate change and a sizeable influence in political developments as compared with 65% in 2017. Examples on well-developed health communications regarding extreme weather events showed that climate change and health are perceived as an important topic in political developments [37, 38].

The need to minimize and prevent adverse climate change-related health outcomes highlights the need for inclusion of health as a consideration in all policies, across all sectors. The 2030 Agenda specifically addresses health (Sustainable Development Goal/SDG 3: Ensure healthy lives and promote well-being for all at all ages) and climate change (SDG 13: Take urgent action to combat climate change and its impacts), as well as a range of targets that support action to protect and promote health through increasing adaptive capacity and health resilience to climate risks, prioritizing mitigation actions that benefit health and pushing the health sector to become less carbon-intensive and more environment-friendly [26]. While responding to climate change is a cross-government priority in many countries, it requires the health sector to work both internally and in a coordinated manner with other actors, often under a single climate change strategy and coordinating mechanism, to define adequate measures. Implementation of the UNFCCC is strongly supported by the 2030 Agenda, which explicitly acknowledges that the UNFCCC “is the primary international, intergovernmental forum for negotiating the global response to climate change”.

The health sector therefore needs to lead adaptation planning for health, working with other sectors to achieve health benefits.

## Health in climate change mitigation

Both air pollutants and greenhouse gases (GHG) are emitted from many of the same sectors, including energy, transport, housing and agriculture. The short-lived climate pollutants such as black carbon, methane and ozone have important impacts on both climate and health. Fossil fuel combustion as the driver of climate change poses a large burden of disease, including a major contribution to the 7 million deaths from outdoor and indoor air pollution annually [7].

The Paris Agreement on Climate Change identifies and promotes measures that both mitigate climate change and improve health, for example, by reducing carbon emissions, air pollution and the environmental impact of the health sector itself [25].

Countries in the WHO European Region have made very substantial commitments to reducing their GHG emissions. The combined commitment of the 53 Member States is equivalent to reducing overall GHG emissions in the region by 26% by 2030, estimated in comparison with baseline emissions in 1990 [41]. Most have set targets to reduce carbon emissions below 1990 levels, while others have set emission caps or intend to reduce future emission growth rates relative to a “business as usual” scenario. Further reductions could be achieved through international cooperation, knowledge sharing and financial support.

Most measures and policies to reduce GHG emissions can benefit human health, if adequately designed and implemented. Carbon-cutting policies that are known to provide health benefits include those that reduce emissions of health-damaging pollutants through changes in energy production, energy efficiency, sustainable transportation and control of landfills, among others.

These commitments are reflected in Member States’ official submissions to the Secretariat of UNFCCC as INDCs, which reflect countries’ ambition to reduce emissions, given their capabilities and circumstances. The annual preventable premature mortality could amount to 138,000 deaths across the whole WHO European Region, of which 33% (45,350 deaths) would be averted across 28 countries of the European Union in 2030 and beyond. In economic terms, the benefit of reduced emissions is equivalent to a savings of 244–564 billion US dollars, or 1%–2% of the WHO European Region gross domestic product (at purchasing power parity prices). The saved costs of illnesses (34.3 billion US dollars) amount to between 6% and 14% of the total economic benefit [41].

## Conclusions

The protection of health from the effects of climate change has developed from a niche topic to high-level policy attention, as reflected in international agreements such as the Paris Agreement and the 2030 Agenda for Sustainable Development. Increasingly, the call to integrate health into all policies and the need to consider climate change in all policies are being recognized and implemented. Understanding and awareness of health risks from climate change is growing fast within the health community; this needs to be reflected as core elements in training and career development for health professionals. Capacity-building is supported through the setting of norms and standards, the development of technical guidance and training courses and the mainstreaming of climate change and health topics into medical and public health training.

The health sector can support and inform policy-making towards the full potential of healthy mitigation through intersectoral action, advocacy, health impact assessment, identifying health co-benefits and win–win policy options and leading by example in reducing its own carbon emissions.

WHO aspires, among others, to support national, regional and global advocacy, provide evidence through country profiles and business cases for investment, ensure technical and capacity-building support for implementation and support climate-resilience, energy and water access in health care facilities. The WHO thirteenth general programme of work (GPW13) is woven around three strategic priorities, each setting a goal of 1 billion people and collectively known as the “triple billion” goal. These include: 1 billion more people benefiting from universal health coverage, 1 billion more people better protected from health emergencies and 1 billion more people enjoying better health and well-being. GPW13 highlights the importance of addressing climate change and health, specifically in small island developing states and other vulnerable settings, and of strengthening cross-sectoral collaboration towards health in all policies. To this end, WHO aims “to ensure that health systems become resilient to extreme weather and climate-sensitive disease” and “to help countries to ensure that global carbon emissions are falling so as to bring health ‘co-benefits’” by 2030 [32].

The draft WHO global strategy on health, environment and climate change, which is to be considered by the Seventy-second World Health Assembly in May 2019, aims to support GPW13 in providing a vision and way forward on how the world and its health community can respond to environmental health and climate change risks and challenges up to 2030 [42]. In the WHO European Region for the priority area of climate change and health, the Ostrava Declaration on Environment and Health calls upon “countries to strengthen adaptive capacity and resilience to climate change-related health risks, to support measures to mitigate climate change and to achieve health co-benefits in line with the Paris Agreement”. To achieve these objectives and planned ones in the forthcoming strategy, countries can include in their national portfolios some proposed actions listed in Table 2.

**Table 2:** Strategic objectives in the draft WHO global strategy and actions to advance implementation of the Ostrava Declaration. (WHO [42] and WHO Regional Office for Europe [29])

Strategic objectives for the transformation needed outlined in the draft WHO global strategy on health, environment and climate change strategy	Possible actions to advance the implementation of the Ostrava Declaration
Primary prevention: to scale up action on health determinants for health promotion and protection in the 2030 Agenda for Sustainable Development, including on the drivers of environmental risks to health	To develop and implement a national strategy or action plan for public health adaptation to climate change as an independent policy or within wider national adaptation policies, as well as natural disaster risk reduction policies
Cross-sectoral action: to address determinants of health in policies in all sectors and ensure healthy energy, transport and other health-determining transition to gain the health co-benefits of environmental protection	To assess climate change risks to health in relevant national policies, strategies and plans
Strengthened health sector: to strengthen health sector leadership, governance and coordination roles in working together with other sectors with relevance to health, environment and climate change	To include, on a voluntary basis, health considerations within Member States’ commitments to the UNFCCC
Building support: to build mechanisms for governance as well as political and social support, including multilateral and other high-level agreements that tackle major driving forces and global threats, such as climate change	To consider climate change adaptation and mitigation in the development of specific environment and health policies, such as those on air quality, water and sanitation and others, bearing in mind that the cornerstones of adaptation include proper health protection infrastructure and housing standards
Enhanced evidence and communication: to generate the evidence base on risks and solutions, and to efficiently communicate that information to guide choices and investments	To strengthen natural risk reduction policies and early warning surveillance and preparedness systems for extreme weather events and climate-sensitive disease outbreaks
Monitoring: to guide actions by monitoring progress towards the SDGs	To develop information, tools and methodologies to support authorities and the public to increase their resilience against extreme weather and climate health risks
	To include the health aspects of climate change in education curricula, non-formal education and workforce continuing professional education
	To scale up public communication and awareness-raising campaigns on climate change and health
	To conduct or update national health vulnerability, impact and adaptation assessments of climate change
	To support research on the effectiveness, cost and economic implications of climate change and health interventions, with a particular focus on mutual co-benefits

The health community should be fully engaged in the national intersectoral mechanisms for adaptation to climate change, including contributing to the development of the health components of national adaptation plans, of nationally determined contributions to the UNFCCC and of the national SDG implementation plans.

Vladimir Kendrovski and Oliver Schmoll are staff members of the World Health Organization (WHO) Regional Office for Europe. The authors alone are responsible for the views expressed in this article and they do not necessarily represent the decision or stated policy of the World Health Organization.
